# Correlation between NLRP6 inflammasome and the levels of inflammatory cytokines IL-1b and IL-18 in serum of patients with Meniere's disease

**DOI:** 10.5937/jomb0-55001

**Published:** 2025-07-04

**Authors:** Mengxiao Li, Ying Zhou, Zhibiao Liu, Xin Jin

**Affiliations:** 1 The Affiliated Huaian No.1 People's Hospital of Nanjing Medical University, Department of Otolaryngology-Head and Neck Surgery, Huaian, China

**Keywords:** Meniere's disease, NLRP6 inflammasome, IL-1b, IL-18, diagnosis, Menierova bolest, NLRP6 inflamazom, IL-1b, IL-18, dijagnoza

## Abstract

**Background:**

Meniere's disease (MD) is a prevalent condition in otolaryngology, with its annual incidence rate increasing. Consequently, understanding the underlying mechanisms of MD is of significant importance. The aim is to investigate the relationship between serum levels of interleukin-1b (IL-1b) and interleukin-18 (IL-18), as well as the activation status of NLRP6 inflammasomes, in patients with Meniere's disease and to evaluate their correlation with the severity of the disease, to improve the treatment strategy of Meniere's disease.

**Methods:**

From March 2021 to December 2023, 75 MD patients were selected from the Affiliated Huaian No.1 People's Hospital of Nanjing Medical University for research, and a control group consisting of 75 age-matched healthy individuals was established. Each participant contributed a 5 mL peripheral venous blood sample, which was archived at -80 °C for subsequent analyses. The expression levels of NLRP6 messenger RNA in the blood samples were quantified using real-time fluorescence quantitative PCR methodology. Concentrations of interleukin-1b (IL-1b) and interleukin-18 (IL-18) were measured via enzyme-linked immunosorbent assay (ELISA). Through a comparative examination of NLRP6, IL-1b, and IL-18 levels between MD patients and the healthy controls, the study delved into the potential association between NLRP6 expression and the circulating levels of these two cytokines. In addition, special attention is paid to the differences between unilateral and bilateral MD patients in the above three indexes to evaluate their effectiveness as potential biomarkers for predicting the severity of hearing loss in MD patients.

**Results:**

In individuals suffering from MD, a notable elevation was observed in the peripheral blood expression levels of NLRP6, IL-1b, and IL-18 (p<0.001). A correlation assessment disclosed a positive association between the blood NLRP6 content and both IL-1b and IL-18 concentrations among these patients. Notably, no substantial disparity emerged in the expression profiles of these three biomarkers when comparing unilateral versus bilateral MD cases (p>0.05). Furthermore, patients at advanced stages (III+IV) exhibited significantly heightened levels of NLRP6, IL-1b, and IL-18 compared to their counterparts in earlier stages (I+II) (p<0.001). Receiver operating characteristic (ROC) curve analyses demonstrated that the area under the curve (AUC) for NLRP6, IL-1b, and IL-18 stood at 0.8731, 0.8089, and 0.7838, respectively, suggesting their potential as proficient diagnostic markers capable of differentiating MD patients from healthy controls.

**Conclusions:**

NLRP6, IL-1b, and IL-18 are highly expressed in the peripheral blood of MD patients. NLRP6, IL-1b, and IL-18 can serve as early diagnostic indicators for MD.

## Abstract (sr)

**Uvod:**

Menierova bolest (MB) je čest poremećaj u otorino-laringologiji, a njena godišnja incidencija raste. Shodno tome, razumevanje osnovnih mehanizama MB je od velikog značaja. Cilj istraživanja je bio da se ispita povezanost nivoa interleukina-1b (IL-1b) i interleukina-18 (IL-18) u serumu, kao i status aktivacije NLRP6 inflamazoma kod pacijenata sa Menierovom bolešću i da se proceni njihova korelacija sa težinom bolesti kako bi se poboljšala strategija lečenja Menierove bolesti.

**Metode:**

U periodu od marta 2021. do decembra 2023. godine, za istraživanje je odabrano 75 pacijenata sa MB iz Pridružene bolnice Huaian br. 1 Medicinskog univerziteta u Nanjingu, dok je kontrolnu grupu činilo 75 zdravih ispitanika odgovarajuće starosne dobi. Svaki učesnik je dao uzorak periferne venske krvi (5 mL), koji je bio uskladišten na -80 °C za dalju analizu. Nivoi ekspresije mRNK (mRNA) za NLRP6 u uzorcima krvi kvantifikovani su metodom fluorescentne kvantitativne PCR analize u realnom vremenu. Koncentracije interleukina-1b (IL-1b) i interleukina-18 (IL-18) su određene pomoću enzimskog imunoadsorpcionog testa (ELISA). Upoređivanjem nivoa NLRP6, IL-1b i IL-18 između pacijenata sa MB i zdravih kontrolnih pacijenata, istraživana je moguća povezanost ekspresije NLRP6 i cirkulišućih nivoa ovih citokina. Pored toga, posebna pažnja posvećena je razlikama između pacijenata sa unilateralnom i bilateralnom MB u pogledu ova tri pokazatelja kako bi se procenila njihova efikasnost kao potencijalnih biomarkera za predviđanje ozbiljnosti gubitka sluha kod pacijenata sa MB.

**Rezultati:**

Kod pacijenata sa MB uočeno je značajno povećanje nivoa ekspresije NLRP6, IL-1b i IL-18 u perifernoj krvi (p<0,001). Korelaciona analiza pokazala je pozitivnu povezanost između nivoa NLRP6 i koncentracija IL-1b i IL-18 kod ovih pacijenata. Međutim, nije utvrđena značajna razlika u ekspresiji ova tri biomarkera između pacijenata sa unilateralnom i bilateralnom MB (p>0,05). Nadalje, pacijenti u uznapredovalim stadijumima bolesti (III+IV) imali su značajno više nivoe NLRP6, IL-1b i IL-18 u poređenju sa pacijentima u ranim stadijumima (I+II) (p<0,001). Analiza karakteristika ROC krive je pokazala da je površina ispod krive (AUC) za NLRP6, IL-1b i IL-18 iznosila 0,8731, 0,8089 i 0,7838, respektivno, što sugeriše njihov potencijal kao dijagnostičkih markera za razlikovanje pacijenata sa MB od zdravih pacijenata iz kontrolne grupe.

**Zaključak:**

NLRP6, IL-1b i IL-18 su značajno izraženi u perifernoj krvi pacijenata sa MB. Ovi biomarkeri mogu poslužiti kao rani dijagnostički indikatori Menierove bolesti.

## Introduction

Meniere’s disease (MD) is a prevalent condition in the field of otolaryngology, and its typical symptoms include paroxysmal dizziness, fluctuating low to mid-frequency hearing loss, and tinnitus [1] [2]. Due to the methodological changes between research and diagnostic criteria, the estimated value of reported incidence varies greatly. The incidence of MD in the world is about 3513 cases/100,000 people [3] and increases year by year. MD is commonly seen between the ages of 40–60 and exhibiting a certain degree of familial clustering. Although MD was first reported as early as 1861, its pathogenesis remains unclear. At present, endolymphatic vessel obstruction, endolymphatic absorption disorders, immune abnormalities, inner ear ischemia, viral infections, allergies, and genetic and environmental factors are all related to MD. In addition, diagnosing MD is difficult at the initial stage, and patients may not exhibit all typical symptoms. After treatment, it still manifests as recurrent dizziness and progressive hearing loss, requiring frequent hospitalisation, seriously affecting their mental health and quality of life [4] [5], and causing a socio-economic burden, which has become a global public health problem [6]. Therefore, elucidating the pathogenesis of MD and looking for more accurate treatment directions is of great practical significance.

Recent studies show that immune factors play a vital role in the pathogenesis of MD. Some studies have found that the endolymphatic sac contains immune cells such as lymphocytes, and molecules are related to inflammation or immune response in the lymph of MD patients [7] [8] [9]. At the same time, many macrophages in the stria vascularis and vestibular end organs of the inner ear can secrete proinflammatory cytokines after activation and cause cochlear injury [10] [11] [12]. In addition, the incidence of autoimmune diseases in MD patients is significantly higher than that in healthy people. There are reactive antibodies against inner ear antigens in their serum, and the levels of various autoantibodies increase [13] [14] [15] [16] [17] [18]. Some studies have also found that the activity of peripheral blood mononuclear cells in MD patients is enhanced, and the levels of inflammatory factor IL-6 and tumour necrosis factor-α (TNF-α) in some patients’ serum are increased [19]. With the development of gene technology, it has been found that MD is related to the genetic polymorphism of cytokines and immune-related proteins [20] [21] [22] [23] [24] [25]. These studies suggest the importance of immune factors in the pathogenesis of MD.

Pattern recognition receptors are a key component of the human innate immune system; they are widely expressed in cell membranes, endosomal membranes, lysosomal membranes, and cytoplasm [26]. Inflammatory bodies are a type of multi-protein complex located inside cells, which are a key part of the innate immune system and involve inflammation and immune responses. They are associated with the occurrence and development of various diseases, such as intestinal inflammation, tumours, and liver disease [27]. The NLRC4, NLRP1, NLRP3, NLRP6, NLRP12, and other components of the NLR family are the core components of inflammasomes, and the apoptosis-related spotted proteins, caspase-1 and caspase-11 form a multi-protein complex called inflammasome [27]. The formation of inflammasomes triggers the activation of specific caspases, leading to the cleavage and maturation of interleukin-1β (IL-1β) and IL-18 from their inactive precursor forms. This process facilitates the induction of inflammatory responses and immune reactions as a defence mechanism against infections or cellular damage [28]. At present, the relationship between MD and inflammasomes is not clear. The main purpose of this study is to explore the relationship between inflammatory corpuscles of NLRP6 and serum levels of inflammatory cytokines IL-1β and IL-18 in patients with Meniere’s disease (MD) to provide a new index basis for clinical diagnosis of MD. The specific mechanism is unclear because the immune system is closely related to the pathogenesis of MD. This study will deeply analyse the influence of activation of inflammatory corpuscles of NLRP6 and serum concentrations of IL-1β and IL-18 on the occurrence and development of MD by measuring related biochemical parameters to provide new ideas and scientific basis for MD’s diagnosis and treatment strategy.

## Materials and methods

### Patients

This study selected 75 patients with Meniere’s disease who received treatment at the Affiliated Huaian No.1 People’s Hospital of Nanjing Medical University from February 2021 to December 2023 as the research subjects, forming the MD group, which included 35 males and 40 females. Age (47.12±9.54) years old; 59 cases were unilateral, and 16 were bilateral. The hearing stages were as follows: The first stage (mild hearing loss, defined as an average hearing threshold 25 dB HL) in 8 cases; the second stage (moderate hearing loss, with an average hearing threshold between 26 and 40 dB HL) in 37 cases, the third stage (severe hearing loss, characterised by an average hearing threshold in the range of 41 to 70 dB HL) in 20 cases, and the fourth stage (extremely severe hearing loss, referring to an average hearing threshold exceeding 70 dB HL) in 10 cases.

Inclusion criteria: (1) Meet the MD diagnostic criteria [9]: A. Two or more spontaneous vertigo attacks, each lasting for 20 minutes to 12 hours. B. Audiologically recorded low to intermediate-frequency sensorineural hearing loss in one ear is defined as the occurrence of the affected ear at least once before, during or after the onset of vertigo. C. Fluctuating auditory symptoms (hearing, tinnitus or tinnitus) in the affected ear. D. Another vestibular diagnosis cannot better explain it. (2) No other cochlear or vestibular related diseases; (3) Age 18 years old. Exclusion criteria: (1) Dizziness caused by other reasons (such as vestibular migraine, sudden deafness, etc.); (2) Merge with other major illnesses; (3) There have been cases of acute or chronic infections in the past two weeks; (4) Glaucoma patients; (5) Individuals who have used steroid drugs or antihistamines within the past month; (6) Additionally, a control cohort comprising 60 healthy individuals who underwent physical examinations concurrently was established. None of these controls presented with ear, nose, throat disorders, history of dizziness, or allergic diseases. There were no significant differences in gender and age distribution between the MD and control groups (*P*>0.05). This research adheres to ethical guidelines and has received approval. All participants were fully briefed on the study details and provided written informed consent before enrollment.

### Sample collection

Collect peripheral venous blood samples from all participants in the study, with a sample size of 5 mL per sample. Place the collected blood at room temperature and let it stand for 15 minutes to allow it to coagulate and separate into serum naturally. Subsequently, the partially coagulated blood was centrifuged and operated at 3000 revolutions per minute (r/min) for 20 minutes with a centrifuge radius set at 12.5 cm. After centrifugation, carefully collect the upper transparent serum portion and transfer it to an appropriate container. Finally, store the obtained serum samples in a low-temperature environment of -80 for long-term preservation and subsequent experimental or analytical use. A lymphocyte separation solution was used to separate peripheral blood mononuclear cells.

### Elisa

An ELISA kit (Shanghai Dongcheng Biology Science and Technology Co., Ltd., China) was used to measure the release levels of IL-1β (INS4010102C) and IL-18 (LC4109) in each experimental group. Every experimental step was meticulously executed following the protocols outlined in the reagent kit to uphold the precision and dependability of the outcomes generated.

### RT-qPCR

Extract total RNA from the sample using Trizol reagent. Follow the instructions provided by the manufacturer to ensure that all steps are completed in an RNase-free environment, and use a spectrophotometer to measure the concentration of extracted RNA. A total of 1 μg of RNA was used as the template for cDNA synthesis, which was carried out utilising a reverse transcription kit (K1691, Thermo Fisher Scientific Shier Technology Company, China). The reaction system for PCR is as follows: 3 μL of sterile water without RNAse, 10 μL of SYBR Green Mix positive and negative primers, 1 μL each, and 2 μL of cDNA. PCR cycle parameter setting: pre-denaturing at 95 for 5 minutes, denaturing at 95 for 15 seconds, annealing and extension at 60 for 30 seconds, and repeating steps 2 to 4 for 40 cycles. GAPDH serves as an internal control, determination of NLRP6 mRNA expression relative to GAPDH using 2- ΔΔ CT calculation method.

### Statistics

Statistical analyses were conducted using SPSS version 28.0. Results are presented as mean values and standard deviations (mean ± SD), and group com parisons were performed using t-tests. Additionally, to evaluate the diagnostic capability of NLRP6, IL-1β, and IL-18 in predicting hearing loss severity in MD patients, receiver operating characteristic (ROC) curve analysis was utilised. For this study, statistical significance was set at a *P*<0.05.

## Results

### Comparison of NLRP6, IL-1 β, and IL-18 levels in peripheral blood between MD group and control group

**Figure 1:**
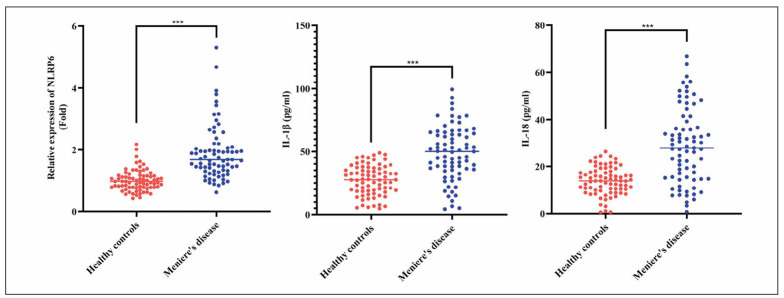
Comparison of peripheral blood levels of NLRP6, IL-1β, and IL-18 between MD group and control group*** p<0.001.

**Figure 2:**
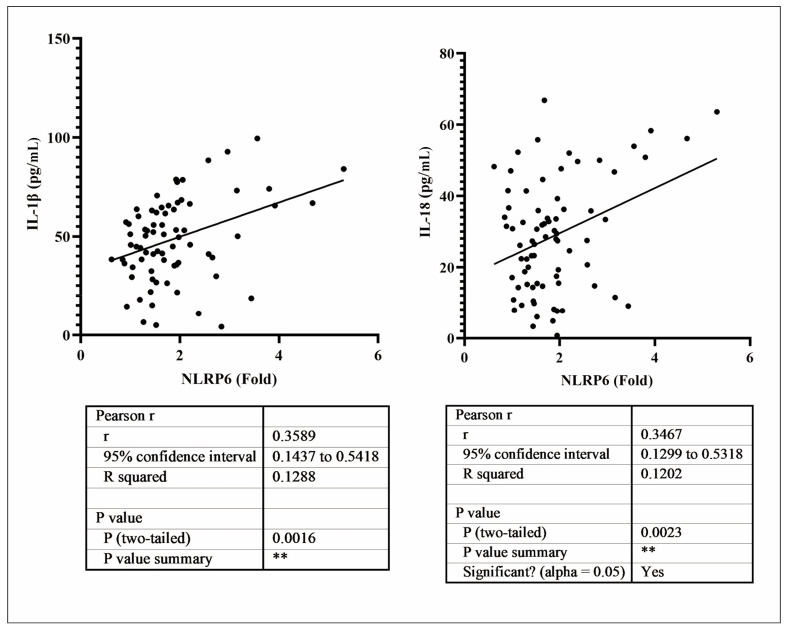
Correlation analysis between levels of NLRP6 and IL-1β, IL-18 in peripheral blood samples.

Firstly, we performed a comparative analysis of peripheral blood samples from the MD and control groups to examine differences in NLRP6 gene expression levels. The results in Figure 1 show that the NLRP6 expression level in the peripheral blood of MD patients was significantly higher (*p*<0.001). Next, we demonstrate that compared to the control group, the MD group exhibited significantly increased expression levels of IL-1β and IL-18 (*p*<0.001), suggesting enhanced activity of these two inflammatory factors in MD patients. Additionally, correlation analysis revealed a positive association between NLRP6 levels and the expression of IL-1β and IL-18, further indicating the potential role of NLRP6 in regulating these inflammatory factors, as shown in Figure 2.

### Comparison of NLRP6, IL-1β, and IL-18 levels in peripheral blood of unilateral and bilateral MD patients

**Figure 3:**
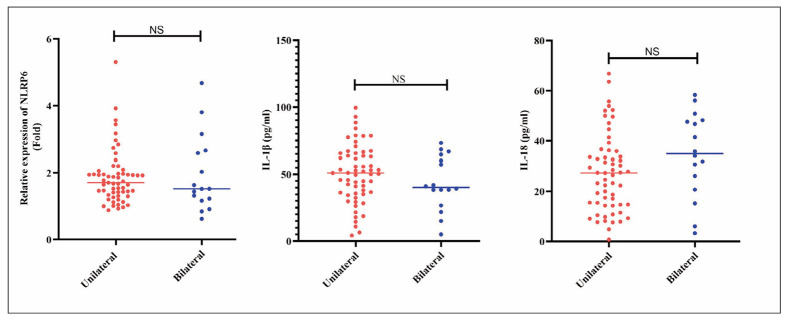
Comparison of peripheral blood NLRP6, IL-1β, and IL-18 levels between unilateral and bilateral MD patients.

Next, we compared the expression levels of peripheral blood NLRP6 between unilateral and bilateral MD patients. The results are shown in Figure 3, with no statistically significant difference observed in NLRP6 expression levels between the two groups (*p*=0.186). Subsequently, we measured the levels of IL-1β and IL-18 of unilateral and bilateral MD patients. The results indicated no statistically significant difference in the release levels of IL-1β and IL-18 between the two groups (*p*=0.158).

### Comparison of NLRP6, IL-1β, and IL-18 levels in peripheral blood of MD patients with different hearing stages

**Figure 4:**
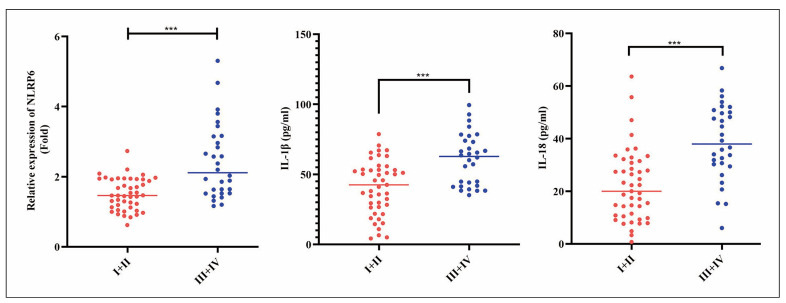
Comparison of NLRP6, IL-1β, and IL-18 levels in peripheral blood of MD patients with different hearing stages. *** P<0.001.

Subsequently, we conducted a comparative analysis of MD patients’ NLRP6, IL-1β, and IL-18 levels across different hearing stages. As illustrated in Figure 4, the NLRP6, IL-1β, and IL-18 concentrations were significantly elevated in patients at stages III+IV compared to those at stages I+II (*P*<0.001).

### The diagnostic value of NLRP6, IL-1β, and IL-18 levels in assessing MD patients

**Figure 5:**
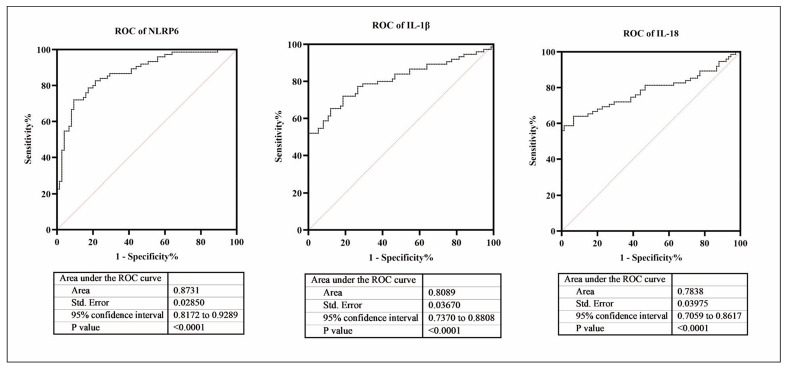
Diagnostic value of NLRP6, IL-1β, and IL-18 levels for MD patients.

Finally, we explored the diagnostic value of MD patients’ NLRP6, IL-1 β, and IL-18 levels. The ROC curve results are shown in Figure 5, where the AUC of NLRP6, IL-1 β, and IL-18 are 0.8731, 0.8089, and 0.7838, respectively. This indicates that NLRP6, IL-1 β, and IL-18 are good diagnostic indicators for distinguishing MD patients from normal individuals.

## Discussion

Meniere’s disease (MD) is a prevalent condition in otolaryngology, with its annual incidence rate increasing. This study investigated the expression levels of NLRP6, IL-1β and IL-18 in patients with myelodysplastic syndrome (MD) and their potential diagnostic value. Previous studies have confirmed that NLRP family proteins, including NLRP6, are key in triggering local autoimmune inflammatory responses in the inner ear, leading to hearing loss [29]. In addition, the levels of proinflammatory cytokines such as IL-1β and TNF-α were increased in patients with macular degeneration, indicating chronic inflammatory diseases [30]. Our study found that the level of NLRP6 in patients with macular degeneration was significantly higher than that in healthy controls. Further analysis showed that patients with advanced disease (III+IV) had higher concentrations of NLRP6 than patients with early disease (I+II), which indicated that NLRP6 was related to the occurrence of macular degeneration and the progressive stage of the disease. In addition, ROC curve analysis showed that the AUC of IL-17/IL-10 ratio used to diagnose hearing stage III or above in MD patients was 0.8731, which indicated that NLRP6 had a high diagnostic value for the severity of hearing abnormality in MD patients. In a word, it is the first time that NLRP6 is elevated in MD patients, and NLRP6 has potential diagnostic value.

NLRP6 can be used as a sensor of the inflammatory body, activating the inflammatory body and identifying various pathogens. It can also recognise other injury signals and secrete and release proinflammatory cytokines, such as IL-1β and IL-18, to induce inflammatory response [31] [32]. It is found that NLRP6 is a key regulator of neutrophil production and recruitment during sepsis after bacterial pneumonia in NLRP6 knockout mice infected with Klebsiella pneumoniae [33]. Xiao et al. [34] found that NLRP6 inflammatory corpuscles may participate in the inflammatory reaction after cerebral haemorrhage by activating microglia IL-1β and IL-18. NLRP6 can indirectly promote the production and secretion of these two cytokines, thus affecting their biological activity level [31]. IL-1β can enhance the inflammatory response, activate local T and B lymphocytes, and up-regulate the immune function of patients, thus worsening the clinical symptoms of patients [35]. In terms of structure and its association with the inflammatory body, IL-18 is the most similar to IL-1β, and both of them have been reported in inflammatory diseases [36], revealing the significant positive correlation between NLRP6 and IL-1β and IL-18 levels, indicating the positive regulation mechanism among NLRP6, IL-1β and IL-18. In this study, we also found that IL-1β and IL-18 increased significantly in MD patients, further supporting their synergistic effect in the occurrence and development of diseases. At the same time, the AUC values of IL-1β and IL-18 were determined to be 0.8089 and 0.7838 by ROC curve analysis, indicating their utility as peripheral blood biomarkers in diagnosing macular degeneration.

Our findings show that the levels of NLRP6, IL-1β and IL-18 in the peripheral blood of patients with macular degeneration are increased. The combined detection of these markers can be used as an early diagnostic index of macular degeneration. However, although this study provides insights into the diagnostic potential of NLRP6, IL-1β and IL-18, it is crucial to acknowledge their limitations. Specifically, this study only analysed the correlation between these markers and macular degeneration activities but did not study the specific regulatory mechanism in depth. In the future, cell experiments and other methods should be used to clarify the precise regulatory pathways involving NLRP6, IL-1β and IL-18 in macular degeneration.

## Dodatak

### Conflict of interest statement

All the authors declare that they have no conflict of interest in this work.
